# Autologous adipose-derived stromal vascular fraction and platelet concentrates for the treatment of complex perianal fistulas

**DOI:** 10.1007/s10151-022-02675-0

**Published:** 2022-09-05

**Authors:** R. Tutino, S. Di Franco, M. Massani, S. Bonventre, G. Mazzola, G. Lo Re, E. Gulotta, L. J. Kamdem Mambou, G. Stassi, G. Cocorullo, G. Gulotta

**Affiliations:** 1 Department of Surgical, Oncological and Stomatological Disciplines (DI.CHIR.ON.S.), University of Palermo, via Roccaforte, 147, Bagheria, 90011 Palermo, Italy; 2 Chirurgia 3, AOU Città della Salute e della Scienza, Turin, Italy; 3 Chirurgia 1, Treviso Regional Hospital, AULSS2 Marca Trevigiana, Treviso, Italy; 4 Unit of Transfusion Medicine, University of Palermo, Palermo, Italy; 5 Section of Radiological Sciences, Department of Biomedicine, Neuroscience and Advanced Diagnostics, University of Palermo, Palermo, Italy; 6 Reconstructive Plastic Surgery, ARNAS Ospedali Civico Di Cristina e Benfratelli, Palermo, Italy

**Keywords:** Perianal fistula, Surgery, Cryptoglandular, Stromal cells, Platelet concentrates

## Abstract

**Background:**

Complex perianal fistulas are a major challenge for modern surgery since 10–35% of patients have functional problems after treatment. Sphincter-saving techniques have a wide range of efficacy (10–80%). We hypothesised that autologous adipose-derived stromal vascular fraction in combination with platelet rich plasma is a new therapeutic strategy with enhanced cure and function preservation rates.

**Methods:**

Adult patients with complex cryptoglandular perianal fistulas were treated with injection of autologous adipose-derived stromal vascular fraction in combination with platelet rich plasma around and inside the fistulous tract between May 2018 and April 2019 at the General and Emergency Surgery Operative Unit of the University Hospital “P. Giaccone” of Palermo. Fistulas were confirmed by magnetic resonance imaging. Patients completed the Short Form-36 score on quality of life and the Wexner and Vaizey scores on faecal incontinence, and they were functionally studied using a three-dimensional anorectal manometry. The clinical and functional follow-up was performed at 1 year and 2 years after surgery.

**Results:**

Nine patients (4 males, 5 females; median age 42 years [19–63 years]) with high trans-sphincteric or horseshoe fistulas were treated. The average number of previous surgeries per patient was 4.8. At 1 year follow-up, 77.7% of patients were cured, while at 2 years there was 1case of relapse. The variation in Short Form-36 score in cured patients was not significant (*p* = 0.0936). No statistically significant differences were found in continence scores.

**Conclusions:**

The proposed treatment is a treatment option that preserves sphincter integrity and function, potentially avoiding postoperative incontinence and the need of repeated treatments.

## Introduction

Perianal fistula is a rare disease (ORPHA:228113), with a prevalence of 1–5/10,000 cases [1, 2]. A complex fistula is usually high, may have multiple external openings, may be recurrent and associated with the presence of pain, fluctuation (which suggests a perianal abscess), rectovaginal fistula, anorectal stricture, and active inflammatory bowel disease [3]. Repeated attempts to cure the patients represent the rule rather than the exception in this disease. An orphan drug has been approved for fistulas related to Crohn’s disease (Alofisel^®^) but no satisfactory treatment has been authorised in the European Union for complex cryptogenic fistulas, which is a major challenge for modern surgery in terms of the integrity of organs, function and quality of life [4].

The aims of fistula treatment are the reduction of symptoms, the prevention of relapses, and the preservation of the integrity of the sphincters and, therefore, of continence [5].

In total, 10–35% of patients treated for complex perianal fistulas suffer from functional problems ranging from soiling to severe faecal incontinence [6].

To avoid such serious consequences/complications, some surgical and non-surgical techniques that preserve perineal integrity have been recently developed. Unfortunately, the healing rates associated with these techniques range from 10 to 80% [7].

The use of stromal cells for the treatment of complex fistulas is a promising area of research, since these cells can facilitate tissue regeneration and healing, thanks to their anti-inflammatory and immunomodulatory effects.

The substrate of anal fistulas is inflamed tissue with faecal contamination [8]. A curative strategy should make use of anti-inflammatory and immunomodulatory factors and growth factor secretion. Autologous adipose-derived stromal cells (ASCs), contained in the stromal vascular fraction (SVF), act by reducing inflammation and promoting tissue regeneration [9]. Autologous platelet concentrates (APC), a source of growth factors and anti-inflammatory molecules, can support and strengthen the role of ASCs, also providing a scaffold [10].

Our research was inspired by previous reports showing through clinical trials that the use of stem cells of mesenchymal origin could be efficiently exploited in clinical settings [5, 11–15]. Indeed, the safety and efficacy of adipose-derived mesenchymal stromal cells in complex perianal fistulas was analysed in a phase I and phase II clinical trial [16, 17].

In phase I, no safety issues were identified. Importantly, biopsies performed in 2 patients showed normal anatomical healing of the infiltrated area [16]. After the first promising results, a phase II study randomised patients to treatment with ASCs in combination with fibrin glue, or fibrin glue alone. In total, 46% of the patients were cured after a single injection of ASCs, and a further 25% after a second dose. Among the cured patients, at 1 year 17% had a recurrence [18]. Unfortunately, the technique did not pass the scrutiny of phase III, in which the treatment was evaluated through a double blind multicentric randomised controlled trial and showed heterogeneity of results between the various centres [19].

However, these studies have been recognised as valid, as they have allowed the development of the drug, Darvadstrocel (Alofisel®), based on mesenchymal cells of adipose origin subjected to expansion. This drug has been authorised for restricted use in the treatment of fistulas in adult patients with non-active/mildly active Crohn’s disease, when fistulas have shown an inadequate response to conventional biological therapy [20, 21].

Of course, cell manipulation involves both ethical and authorisation issues. Under the existing regulatory framework, cellular products that have been subject to more-than-minimal manipulation and/or do not carry out the same function in the recipient as the donor (non-homologous use) are broadly classified as either medicinal products (EU) or biologics (USA) [22]. In this context, we hypothesised that adipose-derived SVF plus APC could represent a new therapeutic strategy for the cure of complex perianal fistulas.

The primary goal of our research was to evaluate the percentage of healing after inoculation of adipose-derived SVF and APC in complex perianal fistulas at 6 months, 1 year and 2 years after surgery. Secondary goals were (i) to evaluate the improvement of clinical parameters in patients with existing anal incontinence using validated scores at 1 year and (ii) to assess the improvements achieved in quality of life (QoL) of patients 1 year after surgery.

## Materials and methods

A prospective study was conducted on adult patients (> 18 years old) with complex perianal fistulas of cryptoglandular origin between May 2018 and April 2019 at the General and Emergency Surgery Operative Unit of the University Hospital “P. Giaccone” of Palermo. Exclusion criteria were: simple fistulas, rectovaginal fistulas, pregnancy, Crohn's disease and ulcerative colitis, hepatitis C virus infection, hepatitis B virus infection, human immunodeficiency virus infection, neoplasms, autoimmune diseases, tuberculosis, psychiatric disorders, and treatment with immunosuppressive or cytotoxic drugs. The present clinical study was authorised by the Palermo 1 Ethics Committee in the session of 17/01/2018 n. 01/2018.

Patients underwent a proctological evaluation including medical history, clinical exam and proctoscopy. Patients with perianal fistulas deemed to be complex were offered inclusion in the clinical trial, and those who accepted completed a QoL questionnaire, the SF-36 (Italian version) [23], and two faecal incontinence questionnaires, the Vaizey score [24] and the Wexner score [25].

**Fig. 1:**
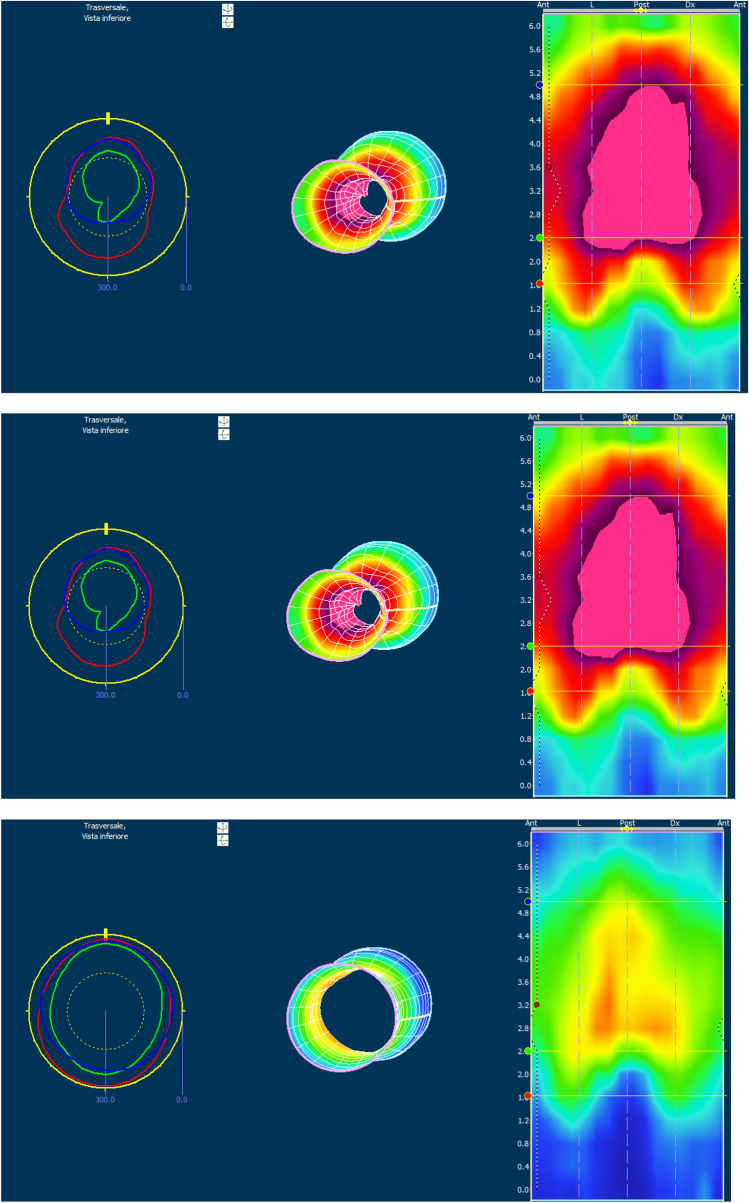
High-resolution and high-definition anorectal manometry images from a treated patient

The clinical evaluation was confirmed using pelvic magnetic resonance imaging (MRI) with contrast medium. Transanal ultrasound was not available. The functional evaluation was completed by the execution of high-resolution and high-definition anorectal manometry) that provides a detailed topographic and colorimetric mapping of the anorectal sphincter complex and offers a more intuitive evaluation of the anorectal function without the need for pull-through of the catheter. This novel technology provides colour-countered topographic plots based on amplitude, distance, and time, depicting a continuum of dynamic pressure changes along lengths and time; data are presented in a simplified manner, in contrast to the use of linear plots of amplitude signals alone in conventional manometry [26] (Fig. 1). Patients with confirmed complex fistulas were treated with adipose-derived SVF and APC after giving their informed consent in accordance with the Helsinki Declaration.

Patients had pre-treatment seton placement and collection drainage for 3 months and were then re-evaluated. In our series, blood samples were obtained from patients for the production of APC up to 7 months before the treatment. We produced 10 ml of APC for the treatment, 5 ml for any treatment of relapses and 2 ml for further laboratory analysis which have been frozen at – 40 °C. The treatment was composed of an abdominal phase, a bank phase, and a perineal phase on the same day.

In the abdominal phase, adipose tissue collection was performed by a plastic surgeon, using a 10 Gauge cannula connected to a 10 ml syringe, after infiltration of the subcutaneous space with a modified Klein solution [27, 28]. 125 ml were taken from each hemi-abdomen. After retrieval, the lipoaspirate was decanted into 50 ml tubes for approximately 5 min and the liquid precipitate containing Klein's solution was removed. The insulated fat was subjected to double centrifugation until obtaining a stromal vascular fraction containing ASCs [29], which was resuspended in the autologous APC. For the surgical procedure, we used the fresh non-manipulated SVF. Moreover, part of the SVF was used to isolate, expand and characterize the ASCs from each patient.

The perineal phase followed the indications proposed by previous studies on the use of ASCs in the treatment of perianal fistulas [30]. The fistulous tract was identified and debrided. The internal orifice was closed with stitches and its closure verified with water injection. After that, we injected the SVF resuspended in APC along the fistulous tract, in the submucosa at the internal orifice, and also directly inside the fistulous tract, in a gelled form. The gel was achieved through the addition of batroxibin plus calcium, to obtain fibrin activation. A gauze with paraffin was placed over the external orifice.

Fresh ASCs were isolated and cultured as previously reported [14, 31, 32].

Follow-up was performed at 1 and 6 months, 1 year and 2 years after surgery. Anal fistula healing was defined as complete healing of the anal wound by epithelization without a residual tract, external or internal openings, or perianal discharge.

### Statistical analysis

Descriptive data are presented as percentage, mean and standard deviation for parametric data, and median, range and confidence interval for non-parametric data. The relationships between age, fistula characteristics, sex and healing were calculated using the Fisher exact test. The comparison between pre- and post-operative scores was performed using the *T* test or Wilcoxon's rank test for paired data. A *p* value < 0.05 was considered statistically significant, and < 0.1 weakly significant. Statistical analyses were performed using MedCalc for Windows, version 19.4 (MedCalc Software, Ostend, Belgium).

## Results

Nine patients (4 males, 5 females; median age 42 years [19–63 years]) completed the preliminary stages and were treated with the ASCs plus APC. The fistulas were all complex as defined by the American Society of Colon and Rectal Surgeons (ASCRS) classification [3]. All the fistulas were trans-sphincteric. In total, 55.5% (5 patients) had a recurrent fistula, with the average number of previous surgeries being 4.8. Table 1 shows the characteristics of the treated fistulas. In 5 patients (55.6%), the intervention was conducted under spinal anaesthesia, with the remaining operations performed under general anaesthesia. All patients completed early and late follow-up up to 2 years.

**Table 1:** Patient demographics data, fistula characteristics, healing after treatment with ASCs + PRP

Patient #	Age, years	Sex	Fistula characteristics				Healing			
			Type	Recurrent	Anterior	Number of previous fistula surgeries	1- month FU	6- month FU	1- year FU	2- year FU
1	53	F	High trans-sphincteric	x		4	Yes	Yes	Yes	Yes
2	36	F	High trans-sphincteric	x		13	Yes	Yes	Yes	Yes
3	46	F	Horseshoe	x		4	Yes	Yes	Yes	Yes
4	19	M	High trans-sphincteric		X	0	Yes	Yes	Yes	No
5	36	F	High trans-sphincteric		x	0	No	No	No	No
6	34	M	High trans-sphincteric			0	Yes	Yes	Yes	Yes
7	41	F	High trans-sphincteric	x	X	3	Yes	Yes	Yes	Yes
8	47	M	Horseshoe	x		3	Yes	Yes	Yes	Yes
9	63	M	High trans-sphincteric		X	0	No	No	No	No

*FU* follow-up; *ASCs* adipose-derived stromal cells; *PRP* platelet rich plasma

There were no intra-procedural complications. Thirty day complications consisted of the appearance of minimal inflammation at the external orifice site in 2 patients, which resolved with antibiotic therapy. At one and 6-months follow-up, two patients showed a persistence of secretion through the external orifice (Table 1). At 1 year follow-up, 7 patients (77.7%) were clinically confirmed to be cured, with a re-epithelisation of the external orifice, while at 2 years there was 1 additional relapse (Table 1).

Fistula healing did not show a statistically significant relationship with anterior fistula location (*p* = 0.16), recurrent fistula (*p* = 0.17), the number of previous surgeries (*p* = 0.13), sex (*p* = 1.0), age (*p* = 0.46), or type of anaesthesia (*p* = 0.17).

**Fig. 2:**
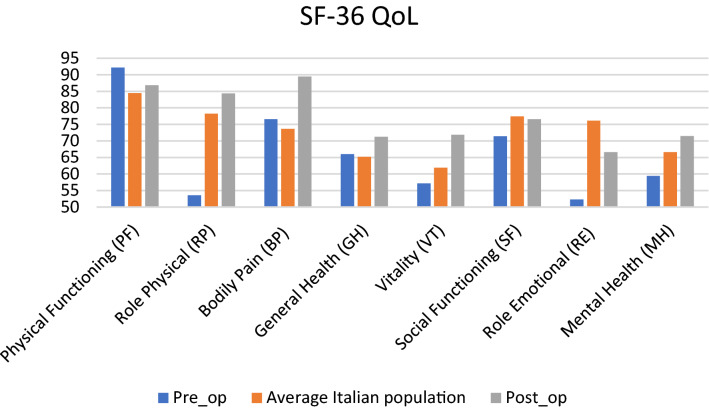
The average Short Form-36 (SF-36) parameters in the pre- and postoperative period of the cured patients compared with the average in the Italian population. *QoL*  quality of life

In terms of QoL, in cured patients we observed an improvement in the general assessment of physical health (PCS) in 5 patients, in 1 patient the value remained unchanged, and 1 patient’s QoL had worsened (*p* = 0.05). There was an improvement in the general assessment of mental health status (MCS) in 5 patients, and a worsening in 2 patients (*p* = 0.17). There was an improvement in PCS + MCS in 5 patients, and worsening in 2 patients (*p* = 0.09). The average SF-36 parameters in the pre- and postoperative period of the cured patients compared with the average of the Italian population are shown in Fig. 2. Although the results were obtained in a limited number of patients, the assessment of the change in health status showed a trend towards improvement after treatment, thus suggesting the need to proceed with this preliminary study, to confirm the obtained data in a larger cohort of patients.

**Fig. 3:**
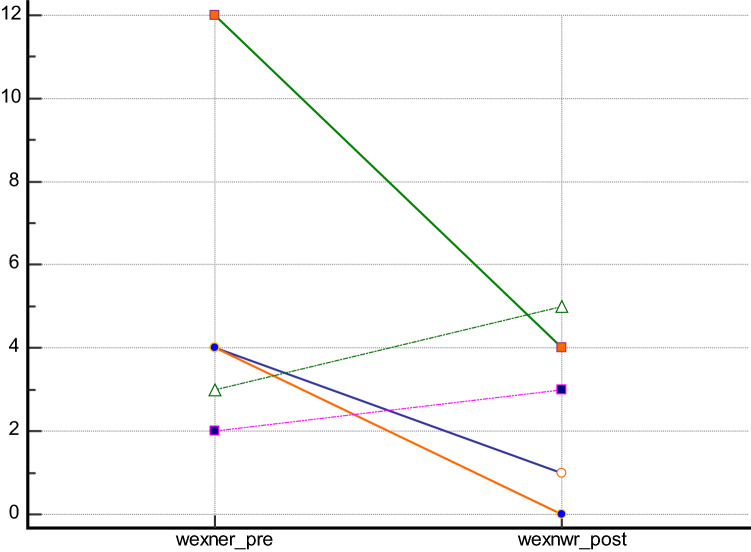
Wexner score changes in patient with previous incontinence

**Fig. 4:**
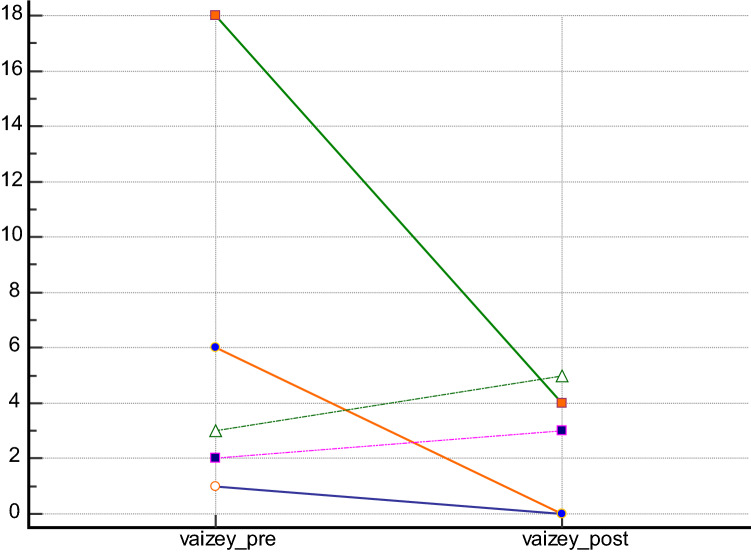
Vaizey score changes in patient with previous incontinence

Regarding the possible improvement of incontinence after treatment, 5 patients suffered from faecal incontinence preoperatively, as assessed by the Wexner [C.I. 2–12] and Vaizey scores [C.I. 1–18] (Figs. 3, 4). Using both the Vazey and the Wexner scores, there was a significant clinical improvement in 3 patients, and a slight improvement in 2 patients. However, the changes were not statistically significant (*p* = 0.31; *p* = 0.44) (Figs. 3, 4).

Flow cytometry analysis confirmed the expression of specific ASC markers, and excluded the expression of endothelial cell biomarkers (CD31) on cells isolated from SVF. The multi-potency of the lipo-aspirated samples was confirmed.

## Discussion

Our proposal is to use non-manipulated ASCs and add a new element, APC, to support the healing process.

Platelet concentrates are potentially useful in wound healing because they function both as a tissue sealant and as a system for the release of growth factors with mitogenic and chemotactic action. Each method of preparation of APC may differ with regard to the number of platelets, platelet activation rates and growth factor profiles [33].

The use of platelet rich plasma (PRP) in the treatment of perianal fistulas was investigated by de la Portilla and colleagues in 2017. They showed a cure rate of only 33% one year after the inoculation of liquid and gelled PRP in a sample of 36 patients. However, the Wexner score in cured patients showed a statistically significant improvement [34]. A prospective multicentre study of 60 patients provided similar results, with cure rates of 40% [34, 35].

More promising results were presented with the use of PRP in combination with mucous flaps, with 26 month cure rates of 80% in a group of 10 patients and 100% in a multicentre study of 25 patients [36, 37].

Since long lasting healing of a fistula should be evaluated over a number of years, in our study, we present the final results obtained at 1 and 2 years of follow-up, showing how the combined treatment with adipose-derived SVF in combination with autologous PRP offers better therapeutic results compared to current treatments with expanded ASCs, achieving a 77% overall healing rate at 1 year and 66% at 2 years.

From the point of view of the possible oncogenicity of the treatment, a published study that retrospectively analysed data on treatment with adipose-derived SVF with a follow-up of 48 months confirmed the safety of the treatment in terms of malignant transformation through examination of histological samples [38]. In the same follow-up study, healing rates at 48 months were reduced to 40%; the authors suggest that the high number of relapses may have been due to the small number of inoculated cells (20 million + 40 million) [37]. Since, we used fresh adipose-derived SVF instead of expanded cells, which is likely to be markedly lower in cell content than the numbers used by other authors, we cannot support the authors’ conclusions.

In the therapeutic protocol, the removal of fibrin and debris resulting from the placement of the seton and a curettage is of particular importance, to create an adequate wound bed for the APC and the en-rooting of stromal cells by exposing vital tissues. It should also be emphasised that the effect of the treatment is local and, therefore, the inoculation should be conducted as close as possible to the walls of the fistulas.

In our treatment, half of the inoculum containing adipose-derived SVF in solution with the liquid APC was injected peripherally to the fistula, while the second half was injected inside the fistulous tract in a gelled form with batroxobin.

Our aim was to create a biological, functionalised scaffold inside the fistulous tract to act as a scaffold for the cells that will repopulate the affected area thanks to the release of the platelet factors contained in the APC.

As for the technique, during the preparation of the surgical field, alcoholic and cytotoxic solutions should be avoided, favouring those based on chlorhexidine [39].

The needle for the injection of the cells should be of a large calibre (i.e., 20–22 Gauge) to avoid mechanical damage to the cells caused by passage through smaller lumens. However, a study has shown that the viability of stromal cells is not compromised by the use of needles up to 26 Gauge [40].

The use of local anaesthesia should be avoided due to the cytotoxic effects of the most commonly used local anaesthetics such as ropivacaine, lidocaine, bupivacaine and mepivacaine. In addition, it has been shown that local anaesthesia can directly and indirectly modify the anti-inflammatory properties of stromal cells in the modulation of macrophages [41, 42].

These notions are critical in the use of adipose-derived SVF, in which high doses of lidocaine are used for liposuction according to Klein's formula. It should be emphasised, however, that a large part of the solution is then removed during the centrifugation of the fat.

We used MRI with contrast in the evaluation and preliminary selection of patients, while the clinical parameter of the absence of secretion through the external orifice, spontaneously and under pressure, with its complete re-epithelialization at clinical evaluation was used to establish their recovery.

The use of the clinical rather than radiological parameters in establishing the healing of a perianal fistula was investigated in a clinical trial in which radiological control of fistula healing after treatment with ASCs showed no correlation with clinical data [38].

The major limitations of the present study are the small sample size and the lack of a control group. Although obtained with a limited number of patients, the results of our study are encouraging, and if confirmed by a larger study, they will support a novel and effective treatment option for patients with complex perianal fistulas.

## Conclusions

The treatment should be performed in reference centres with expertise in the treatment of perianal fistulas, to allow adequate patient selection, and where there is a centre for the production of hemo-derivatives. It can be used with the appropriate indications to offer an advantage in terms of care and possible improvement in the QoL of patients suffering from this chronic and debilitating pathology.
